# How Did This Happen? Xenograft Conversion to Dermal Scaffolding after Scalding Grease Burn

**DOI:** 10.3390/ebj3030035

**Published:** 2022-08-05

**Authors:** Aurelie Tran, Elizabeth Windell, Luke Pumiglia, Amanda Bettencourt, Gary Vercruysse

**Affiliations:** 1 Division of Acute Care Surgery, Department of Surgery, University of Michigan, Ann Arbor, MI 48109, USA; 2 Department of Surgery, Salem Health, Salem, OR 97301, USA; 3 Department of General Surgery, Madigan Army Medical Center, Tacoma, WA 98431, USA; 4 Department of Family and Community Health, University of Pennsylvania School of Nursing, Philadelphia, PA 19104, USA

**Keywords:** xenograft, porcine skin, partial-thickness burns, neodermis

## Abstract

Xenograft and other biologic dressings have been an integral part of burn care for many years. Porcine graft is both inexpensive and, for partial thickness burns, provides the additional benefit of avoiding painful dressing changes when compared with topical agents. In this case, we discuss a patient suffering from deep partial thickness burns for whom xenograft was used for initial wound coverage. This porcine graft became unexpectedly incorporated, and the patient ultimately underwent operative debridement and autologous re-grafting. The case demonstrates a gap in the understanding of wound-healing mechanisms around porcine xenografts and raises the potential for future innovation in expedited wound healing using xenografting.

## 1. Introduction

Several temporary skin substitutes are available for the coverage of partial-thickness burn wounds. Xenografts and allografts have been used for the management of burns for well over a century [1]. Xenografting was first described in the literature in the 1800s and is still used today as a dressing for partial-thickness burns, chronic lesions, and even as an option for temporary coverage of skin graft donor site wounds [2,3,4]. Porcine xenografts provide many advantages for wound care. These include reduction in pain, decrease in the incidence of burn wound cellulitis, and prevention of fluid, protein, and heat losses from wounds. Additionally, xenografts can provide a milieu for autologous re-epithelialization and may facilitate expedited wound healing [1,5,6,7,8]. Xenograft is inexpensive and readily available, and aside from possible reactivity to porcine proteins or zoonotic diseases, is unlikely to cause any significant adverse reactions [1,6,8]. In this case report, we discuss a situation in which the xenograft “engrafted” in the central portion of a burn wound. Experienced burn surgeons have encountered this from time to time, but there are currently few case reports or studies discussing this phenomenon in the literature.

## 2. Case Presentation

**Figure 1:**
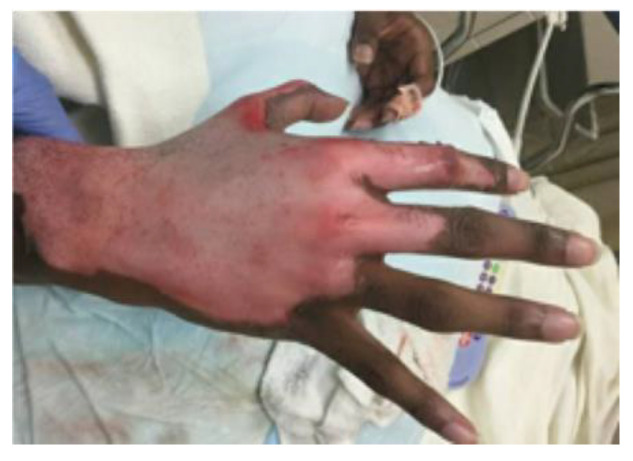
Partial-thickness grease burns to the dorsum of the right hand were superficially debrided in the emergency department.

**Figure 2:**
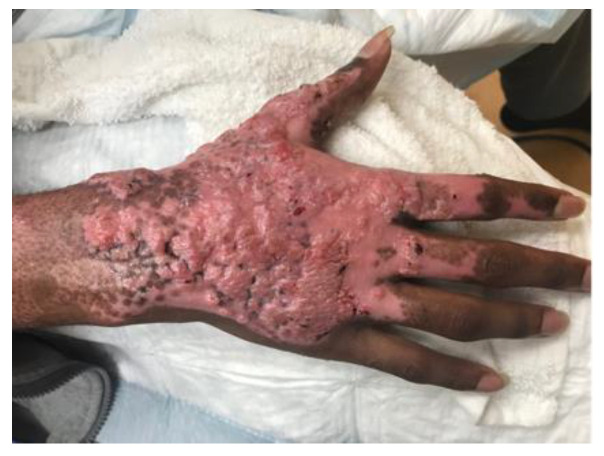
Evaluation nine weeks after porcine xenografting showed an unexpected area of engraftment in the central dorsum of the burn wound with overlying healed epidermis.

**Figure 3:**
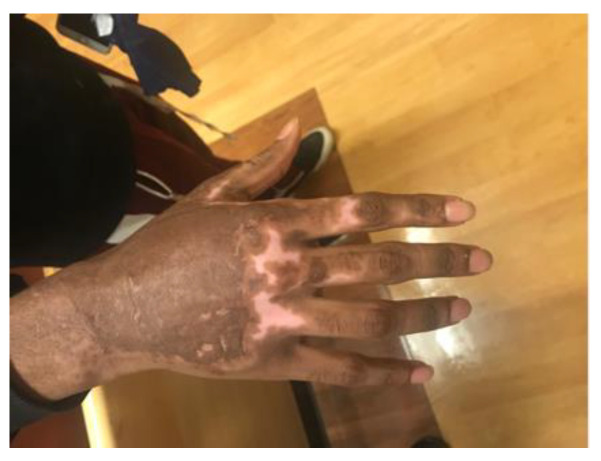
Excision of xenograft with autologous sheet graft coverage was performed ten weeks after his burn. Evaluation three months later demonstrated full healing without contracture or decreased range of motion.

A 15-year-old otherwise healthy boy suffered a splatter grease burn that involved 3% total body surface area (TBSA) to the dorsum of his right hand and foot. The initial burn was superficially debrided in the emergency department, and the patient’s wounds were dressed with 1% silver sulfadiazine cream and cotton gauze (Figure 1). The patient’s wounds were closely monitored for progression. The foot burn showed evidence of healing, but the hand burn progressed from what was initially thought to be a superficial partial-thickness wound to a deep partial-thickness wound despite diligent wound care. He also struggled with severe pain associated with his dressing changes. The decision was made to proceed with xenografting in an effort to help better control his pain by obviating the need for daily dressing changes while allowing the wound to heal under the graft. Informed consent was acquired, and on post-burn day three, the patient was taken to the operating room where, under general anesthesia, he underwent light tangential excision of the eschar overlying the dorsal hand burn. The debrided wound appeared clean, and the exposed dermis and surrounding epidermis appeared viable. As such, no quantitative cultures were taken. Hemostasis was obtained using bovine thrombin and epinephrine-soaked nonadherent dressing, after which the wound was covered with meshed, rolled, lyophilized, porcine xenograft (EZ DermTM, Brennan Medical, St. Paul, MN, USA). It was held with skin staples and nonadherent sterile dressing, used as a circumferential bolster (Telfa ClearTM and Kerlix AMDTM, Cardinal Health Technologies LLC, Dublin, OH, USA; BandnetTM, Derma Sciences Canada Inc., Scarborough, ON, Canada). The patient was discharged home the next day with wound care instructions and oral analgesics. At clinic follow-up on post-burn day 14, most of the xenograft had begun to slough, as expected, revealing normal hypopigmented epidermis. However, an unexpected portion of “porcine engraftment” was noted in the central area of the dorsal hand. Initially, the patient and his parents were averse to reoperation, so the decision was made to closely follow the patient. The xenograft continued to mature with some flattening and was covered with epithelium and closed, but overall was cosmetically unappealing (Figure 2). Ten weeks post-injury, the patient consented to reoperation and underwent a limited excision of the xenograft with autologous sheet graft coverage. At a six-month follow-up, the wound was healed with no hypertrophic scarring and only modest hypopigmentation (Figure 3). From a functional standpoint, he had a full range of motion with no wound contractures.

## 3. Discussion

There are many options for coverage in the case of superficial or deep partial-thickness (second-degree) burns. Simple dressing changes with 1% silver sulfadiazine cream, bacitracin, or gentamycin ointment have been employed for decades. The advantage of these dressings is that they allow for daily inspection of the wound to track progress and monitor the development of burn wound cellulitis.

Unfortunately, these dressings can be very painful for patients and may prolong hospitalization, as parenteral narcotics are commonly required for daily scrubbing and re-application of dressings [5,9]. More recently, silver-impregnated foam dressings have been employed as a reasonable alternative. These dressings have the antimicrobial properties of ionic silver and are well-tolerated once applied. As they can be left on for a week or more, patients can often be discharged after application and will not require dressing changes until clinic follow-up, at which point healing can be assessed.

Alternatively, especially in deep partial-thickness wounds, porcine xenograft can be used after initial debridement as a biologic dressing. Porcine xenograft has been shown to be a useful option in pediatric patients, as it helps reduce pain and the need for procedural sedation [9]. Employing xenograft for partial-thickness scald injuries in pediatric patients can decrease hospital length of stay, reduce the need for reconstructive surgery, and decrease hospital-acquired infections [8,10]. As described in the literature, porcine xenograft, when applied to wounds, will not normally engraft [5,6,8,10,11,12]. Therefore, it is most useful as a temporary covering for partial-thickness burns and may even be employed to cover donor sites or open wounds seen in Stevens Johnson Syndrome or Toxic Epidermal Necrolysis [10,11,12,13]. Once applied, the xenograft will help prevent evaporative water loss, reduce the incidence of burn wound cellulitis, and encourage autologous epidermal growth [5,6,8,11]. It has been posited that porcine tissue allows for a healing milieu through factors released by the porcine tissue [6]. In particular, its affordability, prolonged shelf life, and ready availability make it an excellent alternative to allografting under appropriate circumstances [5,6].

It is necessary to appreciate the process that allows for true “take” or “engraftment” when adherent dressings are placed onto skin wounds to understand the phenomenon of xenograft incorporation in this case. When dressings are placed onto a wound bed, adherence occurs via fibrin deposition [4,6]. This adherence enables the protection of the wound bed, reduced evaporative water loss, and improved healing [4,5,6]. Eventually, native epithelialization underneath the graft as well as the immune response to its antigenicity will cause sloughing of the graft approximately 1–2 weeks after placement [4,5,6,7]. Adherence may last longer in critically ill patients, those with large burns, or those with underlying immunodeficiency. This occurs in both xenografts and allografts. The strong adherence of some dressings is often confused with engraftment or take. However, engraftment is defined by collagen deposition and vascularization [4,6]. This is a much more gradual process and is dependent on several factors, such as viability of the wound bed, quality of the graft, infection, graft injury, etc. 

Therefore, although it may appear that the porcine xenograft “engrafted to the wound,” this is unlikely, as it does not contain live cells that would make it viable for neovascularization [4,14]. While there have been cases of permanent viable allograft incorporation, this phenomenon has not been well documented for xenografts [15,16]. It is most likely that the xenograft serves as a scaffold for fibroblasts, providing neodermal growth where the collagen in the porcine dermis stimulates the ingrowth of autologous cells. The porcine collagen eventually undergoes phagocytosis, and the residual neodermis is covered with epidermis over time. Through this mechanism, the xenografts’ scaffold was likely “incorporated” into the tissues similar to other bioprosthetic materials, such as bovine pericardial patches used for arteriotomy repairs or biological hernia meshes [7,17]. 

However, the difference in the wound healing environment in this case that facilitated this process in the central area of injury remains unclear. In previously documented incorporated allograft cases, hypotheses have included the possibilities that allografts in small populations may bear genetic similarity or that burn-induced immunosuppression prevents graft rejection [15,16]. This phenomenon, however, has not been well documented in xenograft placement. Of course, incidental genetic similarity is not possible in xenografting. Given that the patient in this case had a mildly elevated white blood cell count (14,000 cells/cm^3^) with a slight left shift and no prior history of childhood illnesses or infections, we felt that he had a normal immune system at the time of his burn and recovery. As it was not indicated, we did not formally test his immunocompetency. Moreover, the low TBSA and appropriate sloughing surrounding the area of engraftment make injury-induced immunosuppression unlikely. 

One explanation for the inflammation and hypertrophic expression seen in the central area of the wound may be the chemical crosslinking of glutaraldehyde found in porcine xenograft. Such crosslinking with extracellular matrix bioscaffolds is known to induce macrophages to adopt a pro-inflammatory M1 profile rather than the preferred M2 profile, while leading to the ingrowth of native fibroblasts into the xenograft. While this process stimulates phagocytosis of the porcine tissue, it ultimately results in poor long-term wound healing if these glutaraldehyde crosslinks that are formed as part of the processing of the tissue are not phagocytized with the rest of the xenograft [6,12]. While generally not as concerning in xenografts used as a temporary dressing for burn wounds, these findings suggest the use of non-crosslinked xenografts as possibly more suitable in circumstances where neodermal growth without an inflammatory profile is desired [12]. 

In circumstances in which autografting is not possible, such as very large burns with limited donor site, understanding the biochemical mechanisms of this process could be crucial to optimizing and prolonging the viability of xenografts and potentially creating a new, more cost-effective neodermis, which could eventually be retained and overgrafted with thin partial-thickness autograft. The development of an improved alternative is particularly necessary, as EZ Derm has been recalled in the United States due to issues with intermittent heat seal failures of its packaging (U.S. Food and Drug Administration, 2 June 2021). Efforts have been made to develop porcine-derived acellular dermal matrices (XADMs) showing benefits in supporting tissue regeneration and reducing hospital length of stay [12]. It has also been suggested that co-grafting of XADMs with autografts could be effective for full-thickness burns [18]. XADMs have also been successfully employed for other indications, such as rotator cuff tear repairs with little inflammation [19]. Allograft-derived acellular dermal matrices have also been developed, such as Alloderm, but remain limited in use due to significantly high cost [6,7,12]. Other efforts to prolong the viability of xenografts include innovations in genetic modification to reduce immunogenicity, efforts to increase the immune resistance, and various immunosuppressive therapies [7]. These are currently being evaluated and may offer benefit if they can be proven clinically effective. 

## 4. Conclusions

Though there is currently little literature explaining this process, the research that has been published on the natural history of xenograft digestion demonstrates a potential benefit of xenografting if it can be better understood, optimized, and efficiently utilized [7,8]. As demonstrated in this case, xenografts may provide opportunities for epidermal ingrowth and expedited wound healing. Further studies, including histologic analysis of incorporated xenografts (something that, unfortunately, we did not consider at the time of reoperation in this case), may yield greater understanding into the tissue healing environment that facilitates this phenomenon. In the future, if an optimal tissue healing environment could be obtained, xenograft could become not only a highly desirable reconstructive option for temporary coverage but also serve as a much more affordable neodermis that can be overgrafted with cultured, sprayed on, or thin autograft compared to other currently available neodermal tissue substrates.
